# A case report on desmoplastic ameloblastoma of anterior mandible

**DOI:** 10.1186/s13104-016-1961-2

**Published:** 2016-03-16

**Authors:** Narayan Sharma Lamichhane, Qilin Liu, Hongchen Sun, Wei Zhang

**Affiliations:** Department of Oral and Maxillofacial Surgery, Hospital of Stomatology, Jilin University, Qinghua Road, Changchun, 130021 Jilin Province People’s Republic of China; Department of Oral and Maxillofacial Surgery, Bhaktapur District Hospital, Bhaktapur, Kathmandu, 44800 Nepal; Department of Oral Pathology, Hospital of Stomatology, Jilin University, Qinghua Road, Changchun, 130021 Jilin Province People’s Republic of China

**Keywords:** Ameloblastoma, Desmoplastic ameloblastoma, Segmental mandibulectomy

## Abstract

**Background:**

Desmoplastic ameloblastoma (DA) is a rare variant that accounts for approximately 4–13 % of ameloblastoma, displaying significant differences in anatomical site, imaging, and histologic appearance. It has been included in WHO classification of head and neck tumor (WHO-2005) as a variant of ameloblastoma. The tumor resembles benign fibro-osseous lesion for being frequently occurring in the anterior region of jaws as a mixed radiopaque-radiolucent lesion.

**Case presentation:**

We present a case of DA in a 43-year-old female with a painless swelling in the anterior region of mandible. No fluid was evident on fine needle aspiration. A mixed lesion with multilocular appearance was evident on both panoramic radiographs as well as computed tomography scan. An incisional biopsy confirmed it to be a case of desmoplastic ameloblastoma. Segmental mandibulectomy was performed from teeth 35 to 44. The patient is on routine follow-up and is currently free of ailment.

**Conclusions:**

The present case deserves emphasis because of its unfamiliar appearance, potentially aggressive nature and deceptive radiologic appearance maximizing the chances of misdiagnosis. So, the clinician should be alert enough to include desmoplastic ameloblastoma in differential diagnosis of any lesion/growth with mixed radiolucent-radiopaque appearance having ill-defined borders and occurring in anterior maxilla or mandible.

## Background

Ameloblastoma is the second most common tumor, only next to odontoma, has its origin from the odontogenic epithelium. The tumor is considered benign despite of its locally invasive nature. The follicular and plexiform varieties of ameloblastoma are most common, followed by the acanthomatous and granular cell types. Less frequent cellular variants of ameloblastoma are desmoplastic ameloblastoma, clear cell ameloblastoma, basal cell ameloblastoma, keratoameloblastoma, and unicystic ameloblastoma [1, 2].

Ameloblastomas of 0.9–12.1 % have been reported to be desmoplastic ameloblastomas (DA). Desmoplastic ameloblastoma differs from other variants of ameloblastoma in that it is more frequently seen in the anterior region of jaw and its mixed radiolucent radio-opaque appearance is often more representative of a fibro-osseous lesion [3]. Histologically, desmoplastic ameloblastoma is characterized by extensive stromal desmoplasia, dense collagenization with highly variable odontogenic epithelium islands and cords of various sizes.

The true biologic profile of DA is still not well understood due to paucity of adequate samples.

Sun et al. presented retrospective analysis evaluating clinicoradiographic features of 115 cases of DA that were reported in literature from 1984 to 2008 [4]. The scattered data thereafter and literature review shows that about 170 cases have been reported so far [5–15].

This report is an attempt to help the dental community in developing familiarity with the clinical presentation of desmoplastic ameloblastoma and at the same time emphasizing an index of suspicion in recognizing and treating the peculiar aspect of this unusual lesion.

### Case presentation

A 43 years old female of Han ethnic group from Northeast China, farmer by occupation, presented to the Department of Oral and Maxillofacial Surgery, Hospital of Stomatology, Jilin University, Changchun, China with an asymptomatic swelling in her anterior mandible. The swelling had started 1 year previously and since then, there had been a gradual increase to its present size. She denied experiencing any bleeding, pain or sensory changes. She also denied any history of trauma and the past medical, dental and family history was insignificant.

On physical examination, facial asymmetry due to swelling on the left side of the face was noticed. The swelling was oval in shape crossing the midline thereby obliterating the labiomental sulcus.

The swelling had smooth surface with normal overlying skin but stretched. It was non-tender on palpation.

**Fig. 1:**
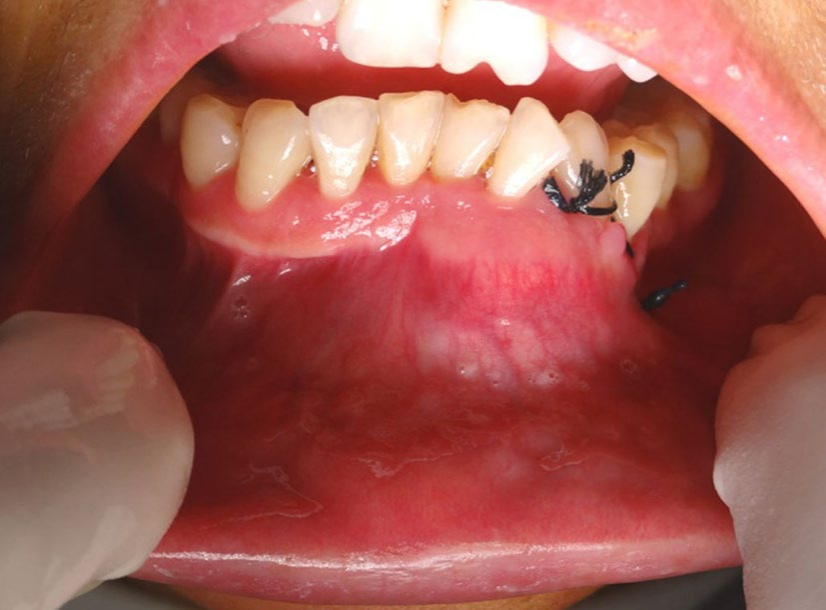
Intra oral view after incisional biopsy

The intra-oral examination revealed a large mass approximately 5 × 4 cm in size, extending from lower right canine to left 2nd premolar buccally. Buccal expansion of the mandibular left and right symphyseal and para-symphyseal region was evident. The overlying mucosa appeared normal. There was labial displacement of 32 (Fig. 1).

On palpation, the swelling was found to be firm, bony hard in consistency, non-tender, non-fluctuant, irreducible, non-compressible and non-pulsatile. The teeth in the vicinity were non-tender to percussion; there was slight mobility of 32 and 33. On electric pulp vitality testing, all teeth in the affected area were vital except 32 and 33. No lymphadenopathy or fistulae were present.

**Fig. 2:**
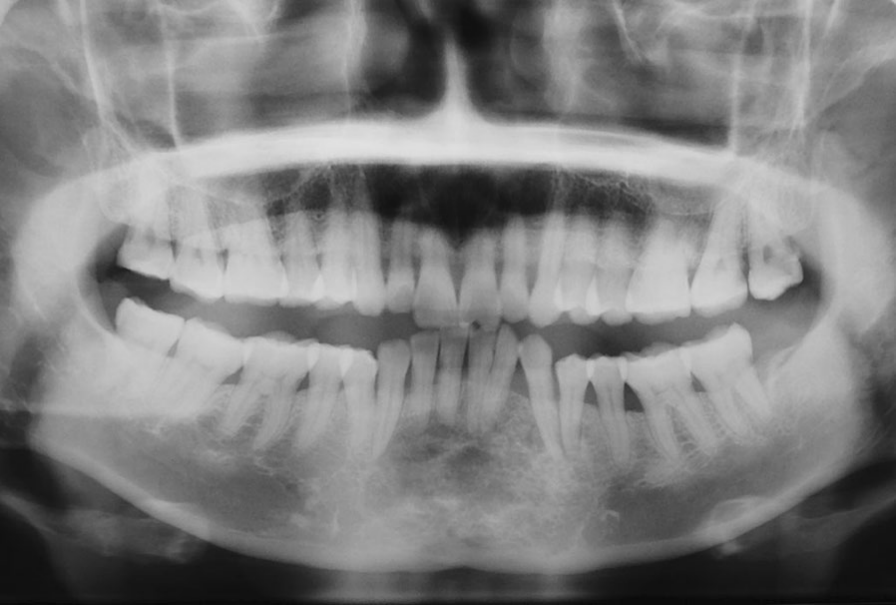
Panoramic radiograph showing mixed radiolucent radio-opacity with ill-defined borders extending from teeth 35 to 43

**Fig. 3:**
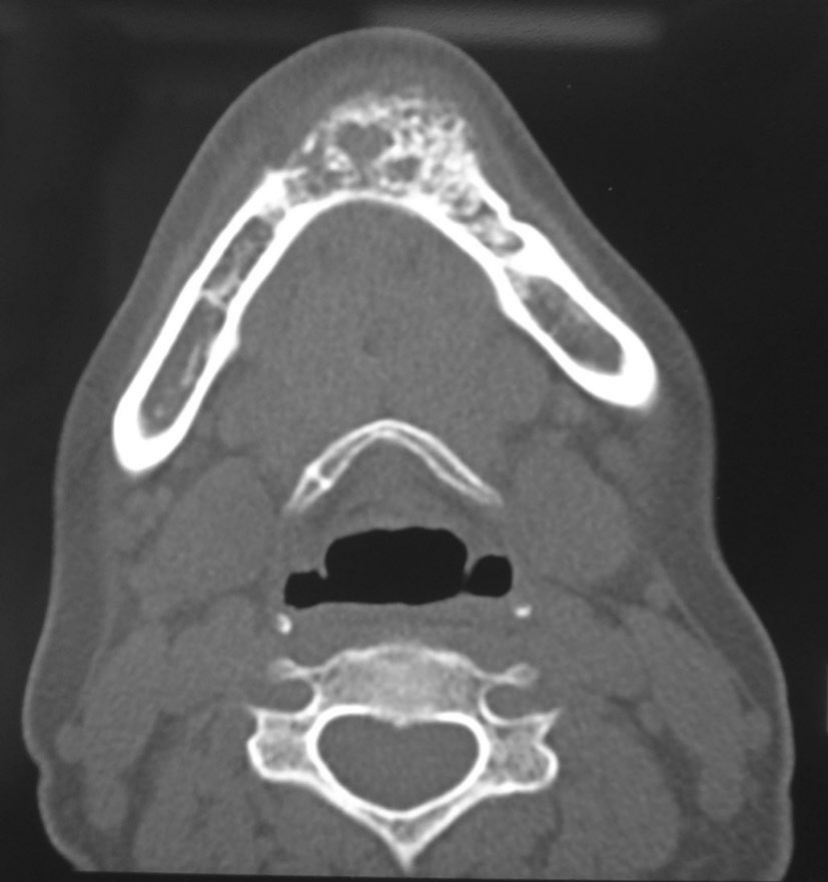
CT scans showing buccal cortical plate expansion

Radiographic examination of the mandible revealed a diffuse ill-defined mixed radiolucent radio-opaque lesion extending from mesial surface of lower right canine to the mesial surface of the lower left 2nd premolar with an approximate size of 5 × 4 cm (Fig. 2). The lesion resulted in the displacement of the roots of 32 and 33 without any signs of root resorption. There was loss of periodontal ligament space on the involved teeth except lower right canine and lower left first premolar. There was loss of lamina dura around the involved teeth. Computerized tomography (CT) of the lesion showed a multiloculated lesion 5 cm mediolaterally, 4 cm superoinferiorly and 2.5 cm anteroposteriorly (Fig. 3). Areas of calcifications were present within the lesion giving it a soap bubble appearance. The lesion almost involved the lower border of the mandible.

A provisional diagnosis of a fibro-osseous lesion of the anterior mandible was made based on clinical and radiographic appearance. The lesion was non-productive on aspiration. Blood profile showed no abnormality except slightly raised levels of serum alkaline phosphatase, 148 IU/L, suggestive of a bone forming lesion. The final diagnosis was established through incisional biopsy performed under local anesthesia. The histologic features were corroborating with those of desmoplastic ameloblastoma.

**Fig. 4:**
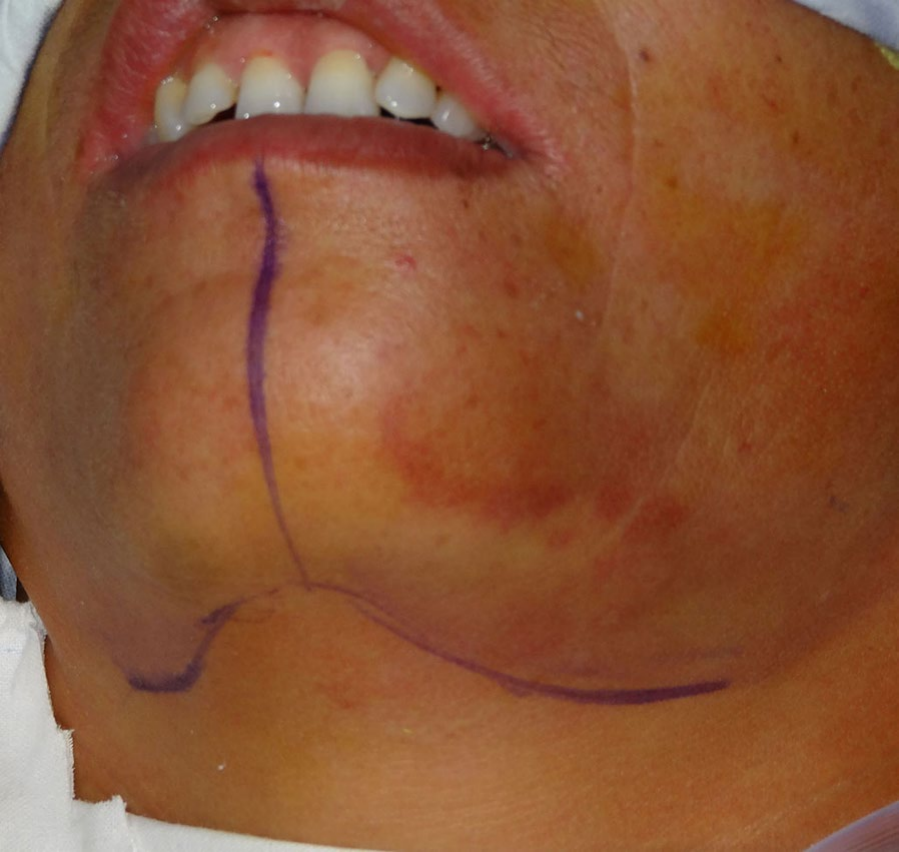
Markings for surgical incison

**Fig. 5:**
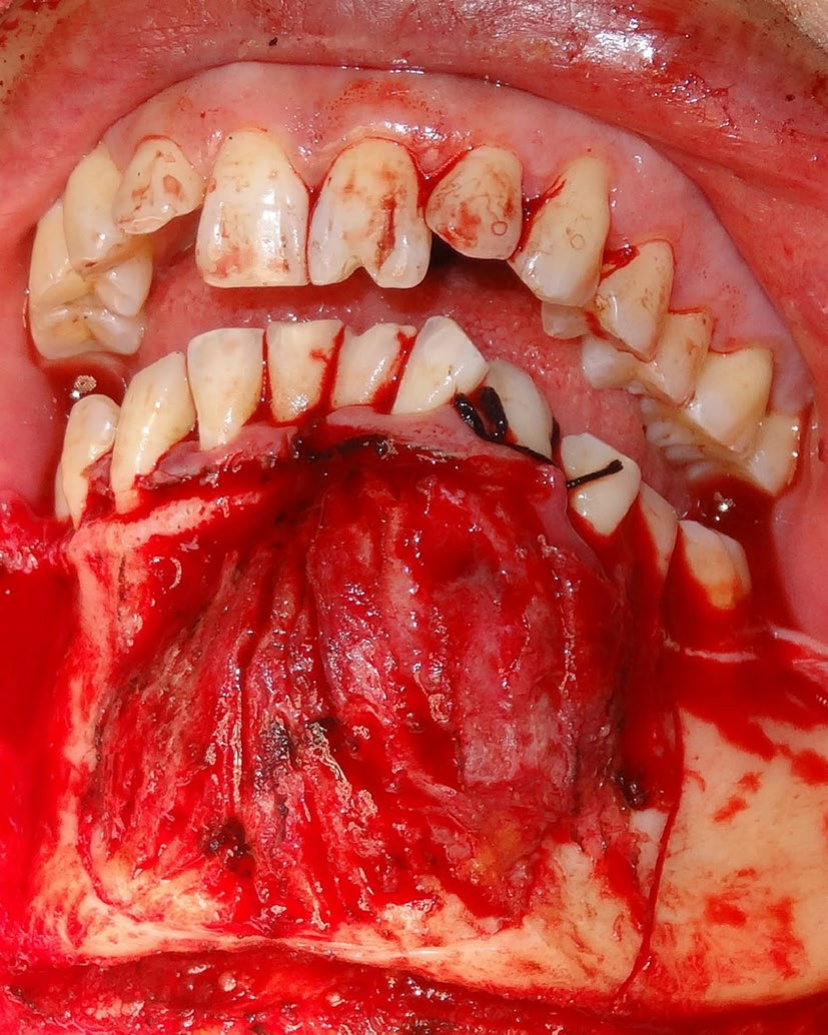
The surgically exposed lesion

A segmental resection of mandible from 44 to 35 was done under general anesthesia with proposed incision as shown in Figs. 4 and 5 and temporarily reconstructed with 2.4 mm reconstruction plate. The surgical specimen consisted of a segment of mandible with the lesion and the associated teeth. The frozen sections were found to be free of tumors. The post-operative period was uneventful. The patient is advised for routine follow-up and has no signs of recurrence so far for a period of 10 months.

## Discussion

In 1984, Eversole [16] pioneered the report on desmoplastic ameloblastoma to the English literature thereby describing three cases and called it an ‘ameloblastoma with pronounced desmoplasia’. In the World Health Organization’s Histopathological Typing of Odontogenic Tumors 2005, desmoplastic ameloblastoma is included as separate clinicopathological entity and classified ameloblastoma into four types as solid/multicystic, extra-osseous, desmoplastic and unicystic [2]. The term “hybrid lesions” was introduced by Waldron and El-Mofty reporting a condition in which desmoplastic ameloblastoma was present in close proximity to follicular or plexiform ameloblastoma [17].

The incidence of desmoplastic ameloblastoma is low. Although DAs are similar to conventional solid ameloblastomas regarding the age and gender distribution, tumors present a strong proclivity for the anterior region with equal incidences on either of the jaw [8]. DA occurs more commonly in the 4th or 5th decades of life, and has no preponderance towards either sex [18]. The mean age of occurrence is 42.3 years (range 17–70 years) [19]. In this report, the age of the patient is 43 years with lesion in the anterior mandible and premolar region, which is consistent with that, reported in the literatures.

The majority of the cases has been reported particularly in Chinese residing in Malaysia and Hong Kong, Malaysians, Japanese and Afro-Caribbeans [20]. Reports from various geographical regions hint at relatively higher frequency of desmoplastic ameloblastoma in Asians [5]. However, more systematic studies on desmoplastic ameloblastoma is necessary to justify such suggestions. Coincidently, the present case of desmoplastic ameloblastoma is also seen in a chinese woman from northeast of china.

According to Philipsen et al., desmoplastic ameloblastomas originating in the maxilla possess more aggressive behavior than those in the mandible. The insidiousness of maxillary lesions to mandibular tumors are attributed to proximity to vital structures as well as the very thin cortical bone of the maxilla being a weak barrier favours the dissemination of tumors. Therefore, maxillary ameloblastomas have potential to spread earlier and more rapidly than mandibular neoplasms.

The extensive presentation of the desmoplastic ameloblastoma may be due to (1) relatively higher incidence in the maxilla leading to an early encroachment of adjacent vital structures (2) the ill-defined diffuse radiographic appearance.

Patient usually presents with a chief complain of painless swelling of the jaw bone. The tumor varies in size between 1.0 and 8.5 cm in diameter [19]. Tooth displacement is a usual finding in desmoplastic ameloblastoma which is seen in approximately 92 % of the cases whereas root resorption is seen in just 33 % of the cases [5]. The patient described in this report also presented with painless swelling with no root resorption, but displacement of the adjacent teeth 32 and 33.

So far as the origin of DA is concerned, Kishino et al. [21] assumed that the desmoplastic ameloblastomas might have originated from periodontal membrane as oxytalan fibers identified in the stromal tissue was stained by potassium monopersulfate-aldehyde fuchsin. Moreover, some others are of the opinion that desmoplastic ameloblastoma may have its origin from epithelial rests of Malassez in the periodontal membrane [22].

Radiographically, about 50 % of DA show a mottled, mixed radiolucency/radiopacity with ill-defined margins, making it difficult to differentiate from a fibro-osseous lesion. It was hypothesized that this may be due to the infiltrative nature of DA to involve the trabeculae. Three radiological presentations of DA are mentioned in the literature as follows: type I (osteofibrosis type) which has radiolucent as well as radiopaque appearance; type II (radiolucent type) which has a completely radiolucent appearance; and type III (compound type) which has radiolucent as well as radiopaque appearance combined with a large radiolucent change [3]. Radiographic features of our case showed mixed radiodensities which were consistent with that of osteofibrosis type (type 1) which is the most common pattern; the compound type is the least common. The lesion is characterized by osseous metaplasia within the dense fibrous septa and this may be the cause for mixed radiographic appearance, it may not be due to mineralized product by the tumor. According to study by Savithri et al. [10] the presumption of newly formed bone rather than destroyed trabecular bone was based on presence of peripheral un-calcified fibrous bone and further added that if tumor cell stimulation of stromal fibroblasts caused desmoplasia, then such stimuli could also have its influence on another cell type, i.e., osteoblasts to stimulate osteoplasia. The present case, too, lacked typical radiographic findings of ameloblastoma and we experienced a little difficulty in diagnosing it correctly. We suspected fibro-osseous lesion or odontogenic myxoma based on the radiographic findings.

**Fig. 6:**
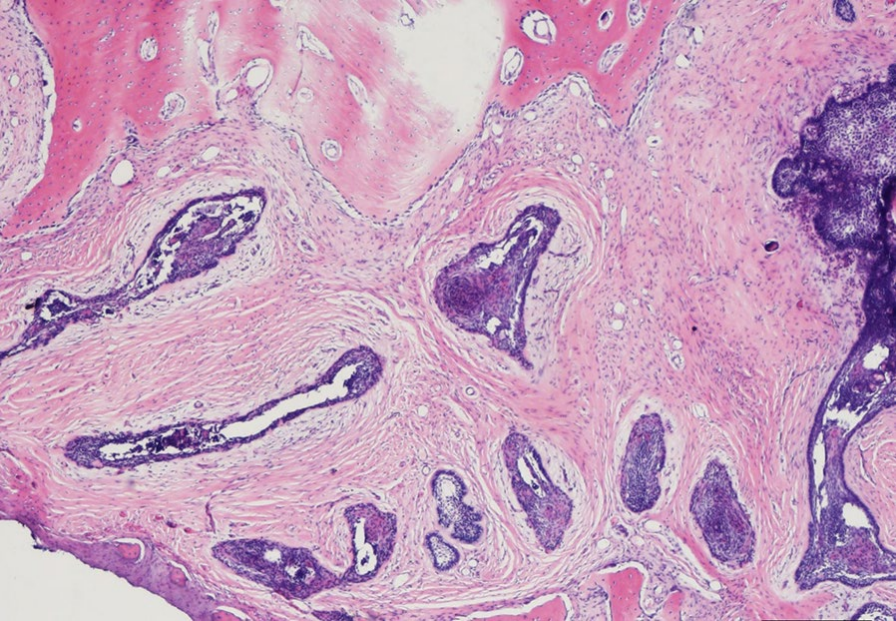
Compressed epithelium surrounded by desmoplasia (×20)

**Fig. 7:**
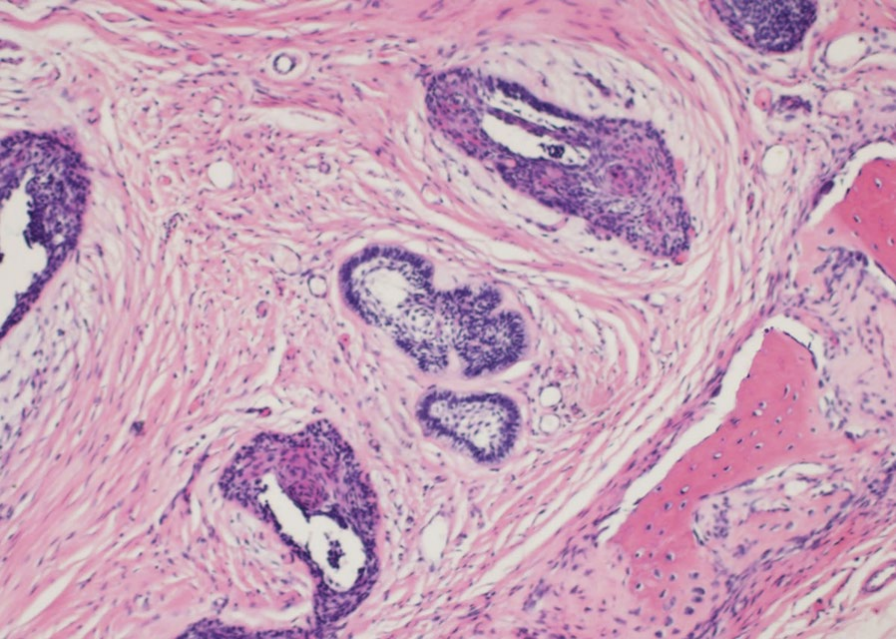
Epithelial islands of different shapes surrounded by desmoplastic stroma (H & E Stain; ×40)

**Fig. 8:**
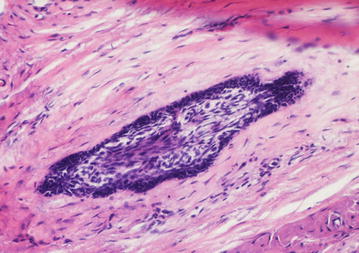
Epithelial island with flattened peripheral cells, and loosely arranged central polygonal and spindle shaped cells (×200)

The final diagnosis of desmoplastic ameloblastoma is based on histopathological evaluation of biopsy specimens. The usual microscopic features are: (1) extensive stromal desmoplasia with abundance of collagen and moderate amount of cellular connective tissue, which is the most consistent and distinguishing feature (Fig. 6); (2) islands of different shapes in the epithelial component (Fig. 7); (3) peripheral layer usually cuboidal and occasionally hyperchromatic; and (4) central area occupied by whorls of spindle-shaped or squamous epithelial cells (Fig. 8). Formation of metaplastic osteoid trabecular (osteoplasia) may be present. The pallisading pattern of follicles as observed in conventional ameloblastoma is absent. Myxoid changes of the juxtaepithelial stroma are often found. A fibrous capsule is not present corresponding to the radiographically poorly defined tumor margin. A DA with features of other histologic variants of ameloblastoma is termed as “hybrid lesion.” Histological findings like intense stromal collagenization or desmoplasia with little odontogenic epithelium islands and cords of various sizes, observed in this case are in agreement to the characteristics presented on literatures.

Histologically, the possibility of misdiagnosis of DA as another odontogenic tumor is high if biopsy specimen is not sufficient enough to warrant the presence of characteristic palisading layer of ameloblastoma in all the epithelial clusters. DA may resemble odontogenic fibroma if presence of only narrow strands of epithelial cells are evident within desmoplastic stroma. The epithelium-poor type of central odontogenic fibroma (COF) is a non-infiltrating lesion of connective tissue that mimics a dental follicle. It contains little cellular component with scattered delicate collagen fibres. The presence of significant quantity of ground substance gives a fibromyxoid appearance to the background in contrast to myxoid changes in the juxtaepithelial stroma in desmoplastic ameloblastoma. Scattered remnants of inactive-looking odontogenic epithelium appear as small irregular islands and cords. Occasionally, variably formed calcifications occur. The epithelium rich type of odontogenic fibroma is composed of cellular, fibroblastic connective tissue that are intermingled with less cellular but vascularized areas. Foci of calcified collageneous matrix mimicking dysplastic cementum, dentin or osteoid are often present. Islands or strands of inactive-looking odontogenic epithelium forms an essential component which may be sparse but are often obvious in contrast to stromal components compressing odontogenic epithelium making epithelial tumor islands very irregular or bizarre with pointed stellate appearance in case of desmoplastic ameloblastoma. The correct diagnosis of these two tumors signifies the clinical behavior and approaches for their management. Ameloblastoma being potentially aggressive tumor requires en bloc resection whereas odontogenic fibroma being much less aggressive requires enucleation.

Squamous odontogenic tumor also comes under differential diagnosis of DA. DA resembles squamous odontogenic tumor if metaplasia of squamous epithelium is evident in some areas but palisading layer of tall columnar cells are not visualized [23]. The islands and strands of DA are often thin and compressed rather than rounded and broad based as seen in squamous odontogenic tumor (SOT). Despite of the aggressive clinical course of some of the SOT, the presently preferred treatment is curettage following which few recurrences may occur. Another differential diagnosis is sclerosing odontogenic carcinoma which is characterized by dense sclerotic stroma containing numerous infiltrating thin cords and small nests of cuboidal or polygonal epithelial cells. The epithelial cells primarily have eosinophilic cytoplasm although some areas of cytoplasmic clearing and a signet-ring may appear. In contrast, DA usually contains large epithelial nests with central spindle cells. Their nuclei show hyperchromatism and slight atypia in contrast to occasional hyperchromastism and no cellular atypia in desmoplastic ameloblastoma. The malignant potential is characterized by encroachement of skeletal muscle and perineural spread. The identification of typical ameloblastic areas forms the basis for definitive diagnosis of DA which requires examination of adequate tissue or repeated biopsy.

Immunohistochemical studies have suggested that the desmoplasia might be a result of overexpression of transforming growth factor beta (TGF-β), a potent local factor that modulates the formation of extracellular matrix. This is attributed to a new protein synthesized from the extracellular matrix that controls cell growth, proliferation, differentiation and apoptosis. Moreover, it has also been proposed that this again synthesis of extracellular matrix proteins involved in the support, adhesion, proliferation, migration and differentiation of tumoral cells might be related to the phenomenon of desmoplasia [24].

Various immunohistochemical studies have supported the fact that DA tumor cells show variable expression of S-100 protein and desmin. There may be increased expression of caspase-3 and Fas(cell surface receptor protein of tumor necrosis factor receptor family), increased expression of p63, decreased expression of cytokeratin19 [3]. Similarly, it has been reported that connective tissue stroma in desmoplastic ameloblastoma displayed a positive reaction for collagen type VI. This was explained as an active de novo synthesis of extracellular matrix protein, hence ruling out scar tissue [5].

Desmoplastic ameloblastoma exhibited desmoplasia of the stromal connective tissue which is thought to be a maturational change in a solid ameloblastoma, as it has been observed that dense collagenisation may be seen in tumors with a long history. This logic can be applied to cases of hybrid tumors. A combination of DA with any other histological type of multicystic ameloblastoma is called a hybrid ameloblastoma. So, it may be hypothesized that a hybrid tumor may be a transitional phase in the maturation of a solid multicystic ameloblastoma to the desmoplastic variety [7].

The biologic behaviour of DA has been depicted in the WHO classification of odontogenic tumors which states DA to have a lower recurrence rate similar to unicystic ameloblastoma and peripheral ameloblastomas. Philipsen et al. reported a recurrence rate of peripheral ameloblastoma with conservative treatment to be 16–20 % [25]. A metanalysis on recurrence rates of intraosseous ameloblastomas of the jaws showed the summary recurrence for solid ameloblastoma to be 8 and 38 % after radical and conservative treatment approach respectively whereas it was 4 and 17 % respectively for unicystic ameloblastoma [26]. A recurrence rate of 21.4 %, which is higher than other type of ameloblastoma (10.1 %) has been reported by Keszler et al. [27]. Having analyzed 115 cases of desmoplastic ameloblastoma from 35 published papers, Sun et al. reported recurrence rate of 21.1 % following enucleation whereas resection decreased this rate to 3.1 % [17]. Thus, recurrence of DA (3.1 %) and solid ameloblastoma (4 %) following resection is comparable to each other.

As the tumor is without capsule, the cells infiltrate between the trabeculae of the cancellous bone leaving them intact for some time. Thus, the tumor actually penetrates beyond the radiographic margin. It could be the possible reasoning for the inconspicuous radiographic margins and the significant recurrence rate following curettage. An analysis of 34 mandibular ameloblastomas by Marx and others reported data showed that the tumor extended 2.3–8.0 mm beyond the radiographic margin [28]. Therefore, they have recommended resection with safety margin of 1 cm of bone beyond the radiographic margin. The reason for recurrence may be somewhat hypothetical: firstly DA commonly presents with inconspicuous margin making the precise interface of the lesion with normal bone cumbersome to investigate. Secondly, the more frequent occurrence in the maxilla may result in an early encroachment of the nearby structures [19].

In view of the paucity of such case series and limited understanding of its biological behavior and prognosis, proper treatment strategies are not completely defined so far. Hence, such cases need to be identified and reported. It is still an enigma whether the recurrence is due to the nature of the tumor (lack of capsule and precise limit) or due to the incomplete surgery. Therefore, resection is the most commonly accepted treatment to prevent recurrence [5]. In our case, we performed segmental resection of mandible with safety margin of 1 cm to avoid recurrence.

## Conclusions

The desmoplastic ameloblastoma is characterized by distinctive clinicoradiographic and histologic features. The clinician must be vigilant regarding the rare presentation of this benign tumor and DA should be included in differential diagnosis of any lesion as simple as abscess to any fibro-osseous lesions or any mass/growth occurring in anterior region of either jaw. Moreover, the horizon of differential diagnosis of Desmoplastic ameloblastoma also extends over any mixed radiolucent–radiopaque lesion with inconspicuous radiographic margin presenting in the anterior–premolar region of the maxilla/mandible. There are still ongoing debates regarding the true biologic behavior of the lesion due to paucity of adequate samples. The radiological and histological findings of poor encapsulation and ill-defined borders suggestive of infiltrative nature warrants in depth analysis and a long-term follow-up. With potential for recurrences, a complete resection to its treatment is recommended alike conventional ameloblastoma.

## Consent

Written informed consent was obtained from the patient for publication of this case report and any accompanying images.

## Abbreviations

DA: desmoplastic ameloblastoma
CT: computerized/computed tomography
SOT: squamous odontogenic tumor
COF: central odontogenic fibroma
